# Supplementary material for: Multimodal MR Imaging for quantification of brain lipid in mice at 9.4T
Source: bioRxiv. 2025 Oct 2:2025.09.30.679612. Preprint. [Version 1] doi: 10.1101/2025.09.30.679612 (PMC12621774; doi:10.1101/2025.09.30.679612)
Supplement: 1 [file NIHPP2025.09.30.679612V1-supplement-1.pdf]

bioRxiv preprint doi: https://doi.org/10.1101/2025.09.30.679612; this version posted October 2, 2025. The copyright holder for this preprint (which was not certified by peer review) is the author/funder, who has granted bioRxiv a license to display the preprint in perpetuity. It is made available under aCC-BY 4.0 International license.

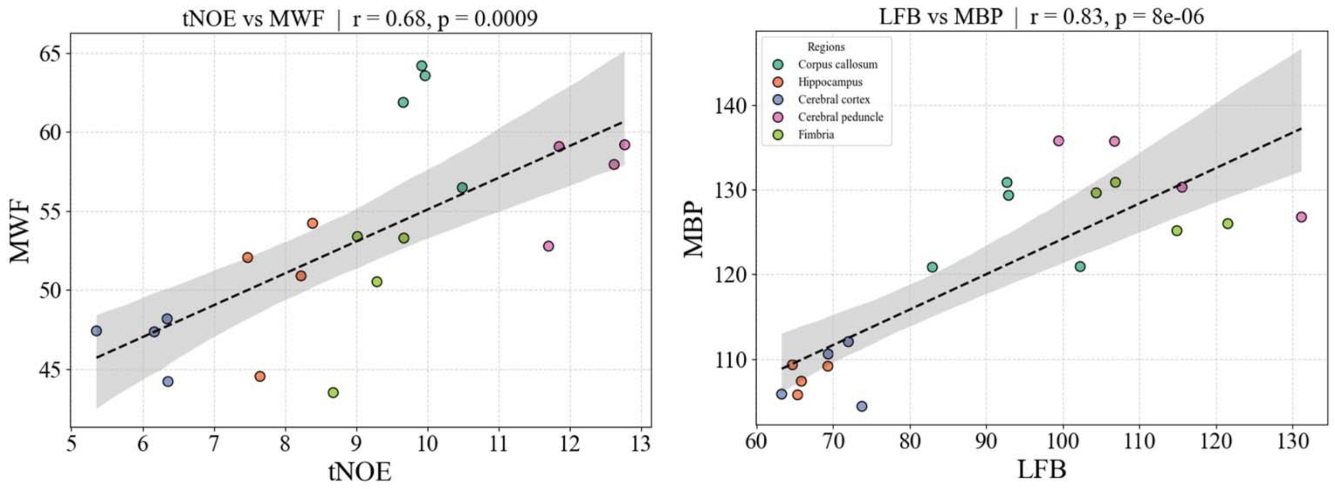

Supplementary Figure 1: Scatter plots display Pearson correlation analyses: A. Imaging-derived metrics from white matter regions, corpus callosum, and cerebral peduncle tNOE, and MWF; B. MBP and LFB from different brain regions.

Supplementary Table 1: Frequency offsets used for NOE acquisition. Fifty-six frequency offsets were applied symmetrically about the water resonance to sample the Z-spectrum for NOE contrast.

[-300; -100; -50; -20; -12; -9; -7.2; -6.2; -5.5; -4.7; -4; -3.3; -2.7; -2; -1.7; -1.5; -1.1; -0.9; -0.6; -0.4; 0; 0.4; 0.6; 0.9; 1.1; 1.2; 1.4; 1.5; 1.7; 1.8; 2; 2.1; 2.3; 2.4; 2.6; 2.7; 2.9; 3; 3.2; 3.3; 3.5; 3.6; 3.8; 3.9; 4.1; 4.2; 4.4; 4.7; 5.2; 6.2; 8; 12; 20; 50; 100; 300]

Supplementary Table 2: Frequency offsets used for tNOE acquisition. Fifty-two frequency offsets were applied symmetrically about the water resonance to sample the Z-spectrum for tNOE contrast.

[-5.006; -4.806; -4.605; -4.405; -4.205; -4.005; -3.804; -3.604; -3.404; -3.204; -3.003; -2.803; -2.603; -2.403; -2.203; -2.002; -1.802; -1.602; -1.402; -1.201; -1.001; -0.801; -0.601; -0.400; -0.200; 0.000; 0.000; 0.200; 0.400; 0.601; 0.801; 1.001; 1.201; 1.402; 1.602; 1.802; 2.002; 2.203; 2.403; 2.603; 2.803; 3.003; 3.204; 3.404; 3.604; 3.804; 4.005; 4.205; 4.405; 4.605; 4.806; 5.006]
